# Optical Micro-Wire Flow-Velocity Sensor

**DOI:** 10.3390/s21124025

**Published:** 2021-06-10

**Authors:** Matej Njegovec, Simon Pevec, Denis Donlagic

**Affiliations:** Laboratory for Electro Optics and Sensor Systems, Faculty of Electrical Engineering and Computer Science, University of Maribor, Koroska cesta 46, 2000 Maribor, Slovenia; simon.pevec@um.si (S.P.); denis.donlagic@um.si (D.D.)

**Keywords:** fiber flow sensor, hot wire anemometer, heat flow sensor, Fabry–Perot interferometer

## Abstract

This paper presents a short response time, all-silica, gas-flow-velocity sensor. The active section of the sensor consists of a 16 µm diameter, highly optically absorbing micro-wire, which is heated remotely by a 980 nm light source. The heated microwire forms a Fabry–Perot interferometer whose temperature is observed at standard telecom wavelengths (1550 nm). The short response time of the sensor allows for different interrogation approaches. Direct measurement of the sensor’s thermal time constant allowed for flow-velocity measurements independent of the absolute heating power delivered to the sensor. This measurement approach also resulted in a simple and cost-efficient interrogation system, which utilized only a few telecom components. The sensor’s short response time, furthermore, allowed for dynamic flow sensing (including turbulence detection). The sensor’s bandwidth was measured experimentally and proved to be in the range of around 22 Hz at low flow velocities. Using time constant measurement, we achieved a flow-velocity resolution up to 0.006 m/s at lower flow velocities, while the resolution in the constant power configuration was better than 0.003 m/s at low flow velocities. The sensing system is constructed around standard telecommunication optoelectronic components, and thus suitable for a wide range of applications.

## 1. Introduction

Measurement of gas/fluid flow velocity using hot-wire anemometers has been used in various industry areas due to their high sensitivity and fast response times [1]. Even though sensors like hot-wire anemometers are well-established in the industry [2,3], fiber-optic flow sensors offer several advantages [4], such as electromagnetic and corrosion immunity, chemical stability, electrical passivity, small dimensions, and the possibility of sensing in remote locations. Optical thermal flow sensors utilize primarily optically heated fiber tips [5,6] or fiber sections [7,8], which also incorporate optical temperature sensors [5,6,7,8]. Many different fiber-optic flow sensor designs have been proposed in recent years, and they are based mainly on spectrally resolved fiber-optic sensors, such as Fiber Bragg gratings (FBG) [7,8,9,10,11,12] or Fabry–Perot interferometers (FPI) [5,6,13,14,15]. Flow sensors that utilize FBGs usually comprise the FBG inscribed into a special optical fiber that contains light-absorbing material at, or in the vicinity of, the grating, and allows for optical induced heating of the sensing (FBG) area. The sensor is heated remotely using a higher-power light source, while simultaneously interrogating the FBG’s peak wavelength. Temperature fluctuations of the sensor are measured through the change in FBG spectral position and correlated to flow. Several different designs were proposed, utilizing special light-absorbing fibers [7,16], silver coatings [17], or graphene layers [9]. Even though the achieved sensitivities are acceptable for many applications, those sensors have relatively long response times due to the considerable diameter (125 μm) of the fiber compared to the diameter of the thin metal wires (<5 μm) used in comparable electrical anemometers. Furthermore, the interrogation of FBGs requires high-resolution spectral analysis of reflected light, which is often costly, making such sensors unattractive for most industrial applications.

All-fiber Fabry–Perot flow sensors offer an alternative to FBG-based sensors. The sensing principle is similar; however, more versatile sensor designs are possible. In most cases, they comprise a cylindrical sensing structure on the tip of an optical fiber [5,6,13], which can be heated remotely using a higher-power light source. Temperature changes are then measured through a shift in the FPI spectral characteristics and correlated to the flow velocity. A general shortcoming of these types of sensors remains similar as in cases of FBG-based sensors, namely, they possess relatively large-diameter sensing structures, yielding longer response times, typically between 140 and 700 ms [6,7]. Furthermore, the contact surface with the optical fiber is often not negligible [5,6,18], and heat dissipation into the lead-in optical fiber should be taken into consideration.

Most current thermo-optic flow sensor designs also assume known or constant optical heating power. However, it is recognized that optical power stability, especially at the end of an optical fiber, can only be achieved and maintained with limited accuracy and long-term stability. Thus, a measurement method that would eliminate the dependence of the measurement results on the heating optical power might contribute significantly to the fiber-optic sensors’ applicability in the broader range of possible applications. Finally, the interrogation systems’ cost remains one of the major obstacles in bringing fiber-optic sensor concepts into industrial and similar applications.

To address the above limitations, we propose an FPI flow sensor formed by a thin micro-wire on the optical fiber’s tip with a diameter of about 16 μm. The sensor’s heated section is formed from a vanadium-doped optical fiber with high optical absorbance at 980 nm. The heated section is further spaced from the lead-in optical fiber by an additional thin section of fiber to prevent heat conduction into the lead-in fiber. Due to the sensor’s small diameter and isolation from the lead-in fiber, its time constant is shortened significantly, and falls into the range between 6 and 2 ms (depending on the flow velocity). The proposed sensor is suitable for static and dynamic flow measurements and has a bandwidth greater than 22 Hz. The sensor’s short time constant also allows for a measurement scheme that relies on the sensor’s time constant measurement rather than on measuring the absolute amount of heat drawn from the sensor by the fluid flow. This provides an opportunity to eliminate the heating optical power amplitude from the measurement result. In addition, we propose a cost-effective interrogation scheme, which does not require a high-resolution spectral analysis, instead relying on a few broadly available telecom components. The paper evaluates the proposed sensor and its performance at static and dynamic flow conditions while relying on two different interrogation schemes.

## 2. Sensor Design and Operation

**Figure 1:**
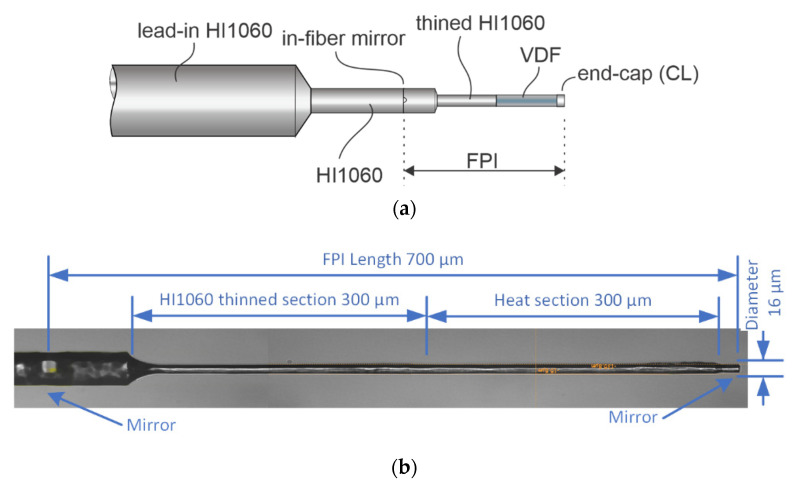
(**a**) Sensor design and (**b**) photograph of the manufactured sensor under a microscope.

The proposed sensor is described in Figure 1. The experimental version consisted of a 125 µm single-mode lead-in HI1060 fiber, a reduced diameter lead-in single-mode fiber section with a length of about 500 µm, which contained an in-fiber mirror near its end facing the flow sensing section, and a flow-sensing section, which consisted of a thinned HI1060 fiber, a thinned vanadium-doped single-mode fiber, and an end-cap. The HI1060 fiber, the thinned vanadium-doped single-mode fiber, and the end-cap had lengths of about 400 µm, 300 µm, and 20 µm, respectively. The vanadium-doped optical fiber (VDF) exhibited high optical absorption at wavelengths around 980 nm, while the absorption within the 1550 nm band remained limited. The optical absorption of the used VDF was about 1 dB/mm at 980 nm, which means that about 10% (300 µm VDF section) of the input optical power was converted into heat at the sensor. This allowed for heating of the VDF section by application of a 980 nm laser source, while providing low optical sensor losses at wavelengths near 1550 nm. The HI1060 fiber section in-between the in-fiber mirror and the VDF, the VDF, and the end-cap formed an all-fiber Fabry–Perrot interferometer (FPI), with a total length of about 700 µm. The diameter of the all-fiber FPI section corresponded to about 16 um, except in a short (about 100 µm long) section beyond the mirror (this short section, which was only reduced in diameter but not fully thinned, is a result of the manufacturing process, shown further below). The entire FPI acted as a temperature-sensitive section.

The vanadium fiber is heated remotely and optically through the lead-in fiber by applying a mid-power shorter-wavelength (980 nm) laser source. Simultaneously, the average temperature of the FPI section is read out through the same fiber using a 1550 nm wavelength band. Small dimensions of the sensor provide a short thermal response time of the sensor. This allows either a conventional sensor interrogation approach, based on King’s law, or the sensor’s thermal time constant measurement. The conventional approach can be realized either by delivering constant power to the sensor while measuring temperature change, or by providing feedback-assisted power control to achieve a constant temperature at the sensor. In the latter case, the delivered power is correlated to the flow rate. On the other hand, the application of the sinusoidal modulation of the heating power can be utilized to measure the sensor’s thermal time constant, which correlates directly with the flow rate and is independent of the heating power and the absolute temperature of the sensor. Within this paper, the focus is given to the second approach due to limitations associated with accurate power delivery in optical systems, as explained in the Introduction. Both approaches are discussed in detail in Section 4.

**Figure 2:**
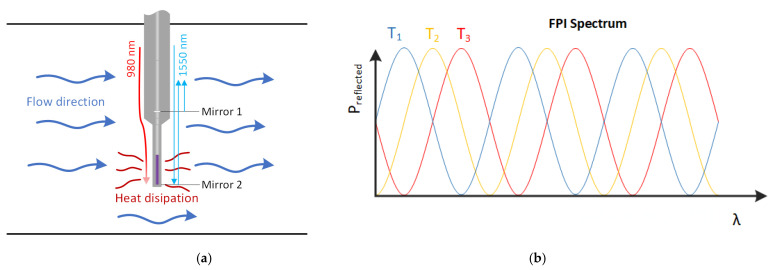
(**a**) Sensor orientation and operation in fluid flow. (**b**) FPI reflected spectrum around 1550 nm, and its shift under a change in temperature (T1 < T2 < T3).

Thus, the fundamental principle is similar to hot-wire anemometers, where the heating power is delivered to the sensing body, which is also a temperature sensor, as presented in Figure 2a. The flow affects both the temperature and thermal time constant of the sensor. In our case, the temperature change is determined from the back-reflected spectrum of the Fabry–Perot interferometer, which shifts with temperature (Figure 2b).

To achieve the short thermal response time of the sensor, which is required for dynamic flow-sensing applications or for efficient sensor operation in an excitation-power-independent mode (as described in the introduction and further below), the radius *r* of the temperature-sensing region shall be reduced to the minimum possible diameter. The time constant *τ* of the cylindrical structure, such as the optical fiber, can be expressed as [19]:

(1) $$\tau =\frac{\rho rc}{2h}=\frac{\rho r^{2}c_{s}}{k_{f}\cdot Nu}$$

where *ρ* represents the density of the sensing section (silica glass), *c_s_* the specific heat capacity of silica glass, and *h* is the heat transfer coefficient, which is expressed further by the fluid’s thermal conductivity *k_f_* and Nusselt number *Nu*. Reduction in the heated section diameter thus reduces the thermal time constant. On the other hand, the sensor’s mechanical/structural integrity must be preserved to allow for robust in-flow operation and resilience to the optical contamination. The latter can be achieved if at least a few micrometers of the cladding is preserved around the heated fiber’s core. As a compromise between these requirements and response time, we thus chose the active region’s diameter of about 16 µm, as already described above in the description of the sensor’s geometry.

After conducting initial experimental measurements of the sensor’s time constants, we concluded that the sole reduction of the heated/sensing section diameter is inefficient for a considerable time constant reduction unless it is insulated from the larger diameter support/lead-in fiber. Especially in gases with low heat conduction, a significant conductive heat flow can be established from the heated section into the lead-in fiber when the thinned heating section is mounted directly onto the tip of a large diameter lead in-fiber. Therefore, we inserted a passive section of thinned single-mode fiber in-between the vanadium fiber and reduced diameter single-mode fiber, as described above and shown in Figure 1. This section acted as a thermal insulating part that limits the direct conduction of generated heat from the thinned VDF into a section of the sensor with a larger diameter. The FPI length was also defined with the end-cap position and the in-fiber mirror, which means that an integral of the temperature change along the entire FPI was detected (i.e., the optical path length change of the FPI correlated to an average difference of the temperature along the FPI). While limiting the sensing region (FPI) just to the VDF region would be optimal from the sensing perspective, the mirror was moved out from the thinned area to the reduced diameter section of the sensor for two reasons: Firstly, it is challenging to produce in-fiber mirrors in the thinned region, since this region was obtained by substantial etching (regardless of the in-fiber mirror creation technique, substantial etching of a splice containing the in-fiber mirror etches at higher rates than pure silica fiber, which leads to the weakening of the splice and subsequent splice failure, i.e., splices containing in-fiber mirrors shall not be etched, especially not over longer periods). Secondly, longer FPIs have a narrower free spectral range, which requires less intense wavelength tuning and adjustments, thus allowing for the introduction of low-cost signal interrogation, as explained in detail in the Signal Interrogation section (Section 4). In our case, an FPI with a length of 700 µm yielded a free spectral range of about 1.2 nm at wavelengths around 1550 nm, which provided different possibilities for integration by using, for example, telecom DFB diodes.

## 3. Manufacturing Process

**Figure 3:**
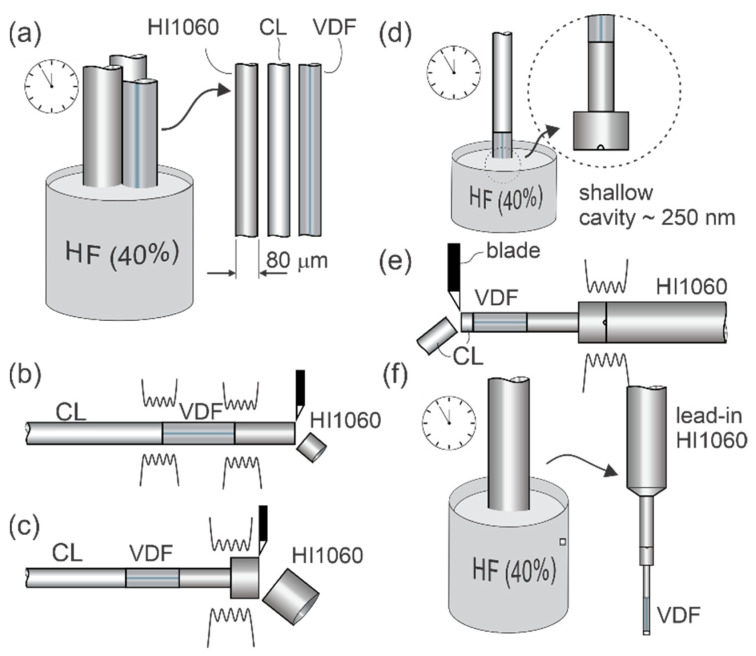
Sensor manufacturing process: (**a**) etching the fibers to the desired diameter; (**b**) splicing the VDF fiber between CL and the HI1060 fiber; (**c**) splicing the sensing section to the lead-in HI1060 fiber and cleaving it for inline mirror manufacturing; (**d**) etching the lead-in section to form a cavity for the inline mirror; (**e**) splicing the etched fiber to the lead-in HI1060 and cleaving the tip of the fiber; and (**f**) etching the sensor to the desired diameter (16 μm).

The sensor was manufactured using several consecutive cleaving, splicing, and etching steps. The entire process is shown in detail in Figure 3.

The sensor production began with the etching of the HI1060 fiber, Coreless (CL), and VDF fiber in hydrofluoric acid (HF), as presented in Figure 3a. All three optical fibers had an initial diameter of 125 μm and were etched for about 23 min in 40% hydrofluoric acid (HF), to reduce their diameters to 80 μm. All three fibers were then used to form the subassembly shown in Figure 3b, where a section of VDF was fusion-spliced in-between the CL fiber and the HI1060. The HI1060 section was then cleaved/trimmed at a distance of about 300 μm from the splice with the VDF (the length of this etched HI1060 defined the length of the thermal insulation part). This subassembly was further spliced to a 125 μm standard diameter (unetched) lead-in SMF, and immediately trimmed about 100 μm away from the last splice on the side of the SMF (Figure 3c). The obtained subassembly was immersed into an HF solution for 45 s. The doped region of the HI1060 was etched at a higher rate than pure silica, which resulted in a shallow cavity at the fiber tip (Figure 3d). The etched HI1060 was further spliced to an HI1060 lead-in fiber (Figure 3e) to create an in-fiber mirror (a technique for mirror production was described in detail in [20]). The sensor’s mechanical assembly was completed by trimming (cleaving) the excess length of CL to about 20 µm. Finally, the entire structure was etched again in HF for about 32 min, as presented in Figure 3f. The obtained structure was immersed 1 mm deep into the HF. The HF surface was covered by a layer of silicone oil to prevent damage to the rest of the lead-in fiber near the sensor. This etching step reduced the outer diameter of the entire HF-submersed sensor assembly evenly. The etching time was selected to reduce the already thinned sensor tip containing VDF and thermally insulting section of the HI1060 to the preferred diameter of approximately 16 μm. The CL fiber on the tip was necessary to prevent etching of the VDF end surface as the vanadium–germanium-doped core etches at a higher rate in HF than the fiber’s cladding. This would degrade the surface at the tip of the structure, used as a semi-reflective surface to create the temperature sensing FPI.

The obtained structure proved to be sufficiently mechanically robust (the thinned sections were relatively short and divided into two sections with different diameters) to allow for easy handling, while still providing short response time thermo-optic flow-sensing responses.

## 4. Signal Interrogation and Modeling of the Demodulation Process

The small diameter of the sensor’s sensing section with a short thermal time constant enables two approaches, which are difficult to realize with fiber-optic thermal flow sensor designs, where fibers with standard diameters (i.e., 125 μm) are used:

(a) Design of high-bandwidth and highly sensitive flow sensors (i.e., a sensor suitable for turbulence sensing and similar applications).
(b) Operation of the sensor in a heating-power-independent regime of operation by applying thermal time constant measurement.

Both approaches utilize the same sensor design, but different interrogation and demodulation processes, as described further below. Thus, the same sensor can operate in any of the above regimes. The sensor’s operation in a heating-power-independent regime of operation is of special interest in fiber-optic sensing systems. Flow sensors based on the heat transfer from the heated body require precise knowledge of the heating power delivered to the sensor, which is a straightforward task in electrical sensors, but it is quite limited in optical systems, especially in fiber-optic systems, where a multitude of influences affect the actual power available at the tip of the fiber (this is, for example, a well-known problem associated with intensity-based fiber-optic sensors). This limitation is often overlooked in the proposed fiber-optic flow sensor designs.

### 4.1. Thermal Time Constant Measurement Flow Sensing (Operation of the Sensor in a Heating-Power-Independent Regime of Operation)

This approach measures a sensor’s thermal time constant rather than the absolute amount of heat drawn from the sensor, thus eliminating the need for absolute temperature and heating power determination. The Nusselt number in the equation describing the sensor’s thermal time constant (Equation (1)) is a fluid velocity function. Therefore, a change in fluid velocity results in a change in the sensor’s time constant. The Nusselt number is a semi-empirical coefficient, which can be approximated for cylindrical structures and low values of Reynolds numbers *Re* as [21]

(2) $$Nu\left(v\right)=0.042Pr^{0.2}+0.57Re{\left(v\right)}^{0.5}Pr^{0.33}$$

where *Pr* is a Prandtl number and *Re* the Reynolds number. The Prandtl number is a function of fluid dynamic viscosity *µ_f_*, specific heat *c_f_*, and thermal conductivity *k_f_*, while the Reynolds number is a function of the sensor radius, fluid velocity *v*, and dynamic fluid viscosity *µ_f_*:

(3) $$Re=\frac{2rv}{\mu_{f}}\;\;\;\;\;\;Pr=\frac{c_{f}\mu_{f}}{k_{f}}$$

Combination of Equations (1)–(3) yields an expression for the sensor’s time constant as a function of fluid flow velocity:

(4) $$\tau \left(v\right)=\frac{\rho r^{2}c_{s}}{k_{f}\left(0.42{\left(\frac{c_{f}\mu_{f}}{k_{f}}\right)}^{0.2}+0.57{\left(\frac{2rv}{u_{f}}\right)}^{0.5}{\left(\frac{c_{f}\mu_{f}}{k_{f}}\right)}^{0.33}\right)}$$

Parameters encountered in Equation (4) for a sensor made from silica glass and immersed in the air at standard conditions are presented in Table 1.

**Figure 4:**
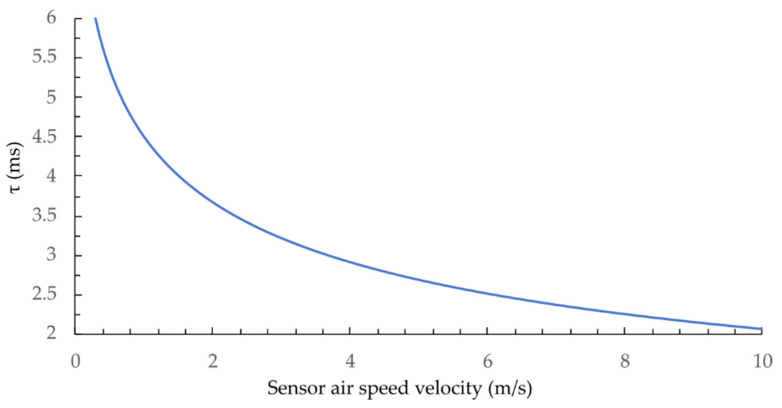
Modeled time constant of a 16 μm-diameter silica micro-wire versus flow velocity in standard air.

Figure 4 represents the calculated sensor’s time constant at fluid velocities between 0.1 m/s and 10 m/s using the parameters from Table 1 and the numeric model (Equation (4)). Results show that the proposed sensor’s time constant falls between 6 and 2 ms for fluid (air) velocities up to about 10 m/s.

As demonstrated by Equation (4), the thermal time constant is independent of the heating optical power. However, it should be stressed that the signal-to-noise ratio (SNR) depends on the delivered heating power; thus, the quality of the measurement signals and resolution will increase with increasing the heating power. The sensor’s time constant can be determined by applying dynamic excitation to the sensor’s heating part while observing and analyzing the time-dependent response.

**Figure 5:**
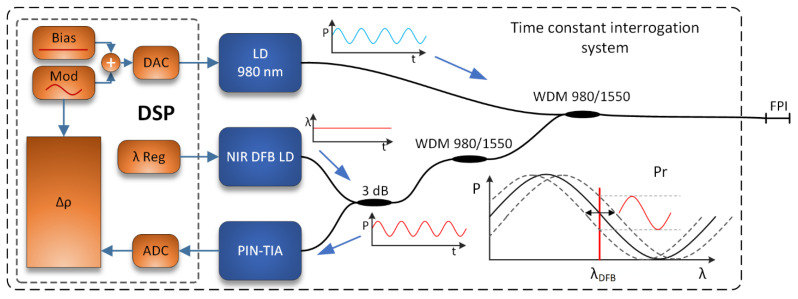
Proposed readout system based on determination of the sensor’s time constant (time constant interrogation system).

The proposed interrogation scheme is presented in Figure 5, and comprises a 980 nm heating laser diode, signal distributed feedback (DFB) laser diode (1550 nm), photodetector with a trans-impedance amplifier, a driver for the heating laser diode, digital signal processor (DSP), and optical couplers (WDM and standard 3 dB), connected as presented in the schematics (Figure 5).

**Figure 6:**
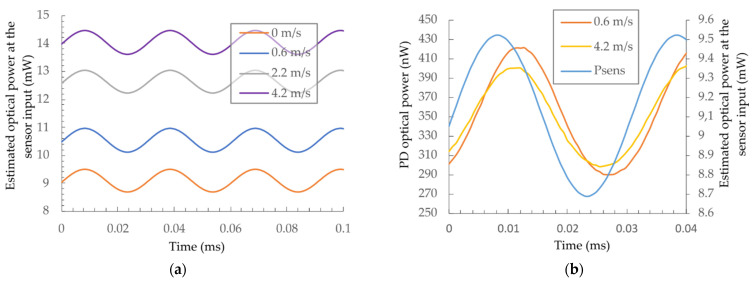
(**a**) Modulation signal at different sensor flow velocities (average power was adjusted by closed loop temperature control to keep the sensing interferometer at its quadrature point), and (**b**) optical power of the photodetector at different sensor flow velocities compared to the modulation signal.

Firstly, the output wavelength (1550 nm) and the power of the signal DFB laser diode were stabilized using the diode’s temperature and driving current control. The sensing FPI was then brought into a quadrature point (the midpoint between the minimum and maximum values of the back reflected operating optical power) at 1550 nm by using the closed-loop control of the heating diode’s output power. A simple initialization algorithm was used, which varied the temperature of the FPI over a broader span and recorded the min. and max. back-reflected optical powers. The mid-point between the recorded min. and max. back-reflected powers were then searched and maintained by feedback-based control of the heating diode’s drive current/output power. This locked and stabilized the average temperature of the FPI during sensor operation. A cosine modulation signal with a frequency of 33 Hz and amplitude of about 1 mW was then superimposed on the heating diode’s bias power/current. Figure 6a depicts the sensor operation at different flow rates. The average heating power is the lowest at zero flow velocity as the heat drawn from the sensor is the lowest. An increase in the flow velocity increases the draw of the heat, which requires more power in order to sustain the same operating temperature of the sensor. In all cases, the superimposed power modulation amplitude remains the same.

This resulted in continuous modulation of the temperature of the FPI, which was recorded by observing the AC component of the back-reflected signal at the wavelength of 1550 nm, as presented in Figure 6b. As the flow rate increases, the time delay between the modulation signal and the signal recorded by the photodetector decreases (which is proportional by the temperature change). The AC component of the signal from PIN-TIA was phase-compared with the initial modulation signal, while the DC component of the signal was fed into the heating diode bias regulation system. The phase difference between the modulation signal and the AC component of the back-reflected signal was obtained using both signals’ simultaneous acquisition and digitalization. During this process, phases of both acquired signals were obtained by application of the Goertzel algorithm [22] and further subtracted to obtain their difference (ΔΦ). The sensor’s time constant was then obtained from the calculated phase difference ΔΦ as

(5) $$\tau =-\frac{\tan\Delta \Phi }{2\pi f};\;-\pi \leq \Delta \Phi \leq 0$$

The obtained time constant was finally used to calculate the flow velocity according to Equation (4) or using the calibration data/function.

To prevent unwanted reflections of the heating 980 nm light into the PIN-TIA, we introduced an additional WDM coupler between the sensor and the photodetector (980 nm wavelength suppression rate was around 20 dB per WDM coupler). All processing of the signals and the closed-loop control were done with a DSP, minimizing external circuits to PIN-TIA, laser drivers, and a TEC controller.

This demodulation method yielded an excitation-power, amplitude-independent operation, while the overall response time of the proposed method was restricted to several full modulation periods, which are required to extract the phase difference between the modulation and reflected signals reliably.

### 4.2. Constant Power Regime of Operation

The sensor’s short response time allows for dynamic flow sensing, such as turbulent flow-sensing applications. The time constant measurements approach described above does not allow for very short response times, since it requires a few modulation signal periods to determine the phase difference between the modulation and readout signals reliably.

**Figure 7:**
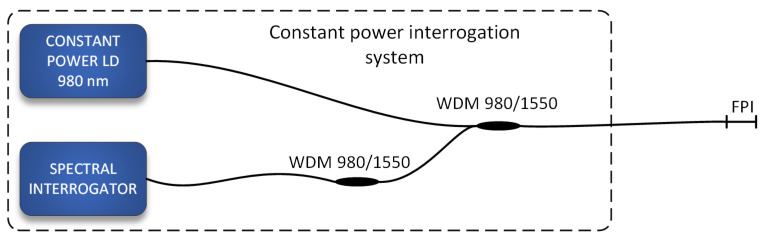
A setup for operation of the sensor for dynamic sensing applications: The sensor is supplied by constant heating power, while the temperature is measured by a fast spectral interrogator and correlated to the flow velocity.

A more conventional approach can be applied in this case, where a constant heating power is delivered to the sensor while observing and correlating the temperature change of the heated sensor body to the flow velocity (i.e., utilizing L.V. King’s law principle). This configuration yields the shortest response times. The interrogation system is presented in Figure 7**,** and consists of a constant power heating laser diode (CP-LD), a high-speed spectral interrogator based on a swept wavelength (SW-LD) laser source [23,24], and a WDM coupler.

The heating laser diode provides constant power to the FPI sensor, while the spectral interrogator tracks the shift of the sensor’s spectral characteristics, which is further correlated to the change in the sensor’s/FPI’s average temperature. Furthermore, the sensor’s ambient temperature is determined by occasional inhibition of heating (this could also be done with a separate temperature sensor), which is required to establish the ambient temperature reference point. The difference in the sensor’s and the ambient temperature is then used as a measure for flow velocity. The flow velocity versus the difference between the sensor’s and the surrounding temperature is obtained through the calibration process, as described further in the Experimental section. The main limitation in the sensor’s dynamic performance is governed by the sensor’s time constant. By defining the sensor’s bandwidth as *f*_3*dB*_ *=* 1/2*πτ* and inserting typical data from Table 1 into (Equation (4)) (also shown in Figure 4), the predicted sensors’ bandwidths fall between 24.5 Hz and 80 Hz, depending on the flow velocity.

## 5. Experimental Setup and Results

The proposed sensor and signal interrogation systems were tested experimentally using the three setups which used the same sensor while applying different interrogation approaches.

### 5.1. Evaluation of the Interrogation System Based on the Time Constant

**Figure 8:**
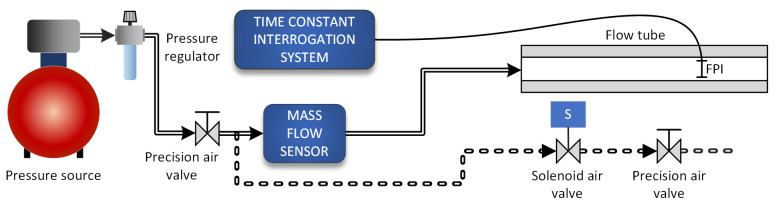
Experimental setup for evaluation of the sensor and interrogation systems based on the thermal time constant measurement.

The setup, used to evaluate the sensor and the interrogation technique based on the thermal–time constant measurement, is illustrated in Figure 8.

The proposed flow sensor was inserted into the center of a 0.6 m-long flow tube with an inner diameter of 7 mm (cross-section of 36 mm^2^). The sensor was located at a distance corresponding to 2/3 of the tube length, measured from the flow inlet to allow for a sufficiently unobstructed flow path before and after the sensor. A precision MEMS mass flow sensor (Omron D6F-10A61-000) was connected in a series with a flow tube for reference airflow measurement. The flow velocity at the sensor *v* was calculated from the average flow velocity obtained from the reference flow meter. The average flow velocity was correlated to the flow velocity at the sensor as *v* = *v_avg_* * (1 + *n*). We assumed *n* = 1, as the sensor was positioned exactly in the center of the tube, and since, in our experiments, the Reynolds number in the tube did not exceed 2300 (a boundary value for a transition from a laminar to a turbulent flow) [25]. An air compressor with a pressure-regulating valve generated working pressure, while a precision air valve connected in series with a pressure regulating valve allowed for precise airflow adjustment (the distance between the valve and the mass flow meter was over 1 m to allow for stabilization of flow conditions). The solenoid valve and additional precision valve were also connected in parallel with the mass flow sensor and a flow tube, to allow for small and rapid flow-velocity changes to determine measurement resolution and the system’s dynamic performance. The electrical control of the solenoid valve enabled rapid step changes in airflow through the flow tube, while the precision air valve connected in series with the solenoid valve allowed for the precise setting of these changes in the flow velocity.

**Figure 9:**
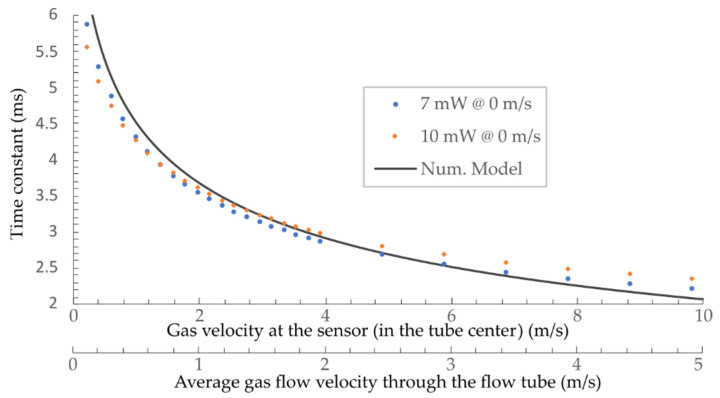
The obtained sensor static characteristic: two measured characteristics at two different quadrature points (temperatures) compared to the simulated results.

The heating laser diode was modulated with 33 Hz, while the average flow velocity through the flow tube varied between 0.1 and 4.5 m/s, meaning that the flow velocity across the sensor varied between 0.2 and 9 m/s (*v_sens_* = *v_avg_* × 2 in the center of the tube). The time constant of the sensor was measured by the interrogation system described in Section 4.1 (Figure 5). The measured sensor’s thermal time constant versus measured preset flow velocity is presented in Figure 9.

The static characteristic was obtained at two substantially different heating operating optical powers/temperatures (i.e., the sensor was locked in two neighboring quadrature points, which required two different average heating powers). The static flow-velocity characteristics recorded at substantially different heating power levels nearly coincided. This indicates that the prosed approach is robust to changes in the transmission loss variations, and almost independent of the operating heating power, which was one of the primary motivations for the presented investigation. Furthermore, experimentally measured time constants were in excellent agreement with the numerically obtained modeling results.

**Figure 10:**
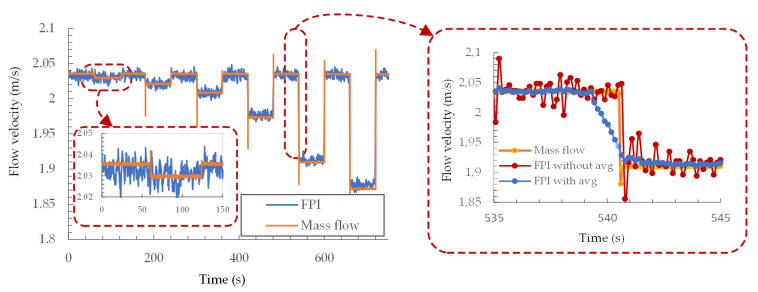
Demonstration of the resolution and response time using incremental flow-velocity steps.

We used a solenoid valve and precision air valve in a by-pass configuration with the flow tube and mass flow sensor, as already described above, to investigate the proposed sensor’s resolution and response time. Using a precision air valve setting, we set the desired flow-velocity change induced by opening the solenoid valve accurately. Rapid solenoid valve opening also created an immediate change in the flow velocity. The flow velocity was then measured continuously by the sensor while we opened and closed the solenoid valve periodically at different precision valve settings to induce a controlled step change in the flow velocity, as presented in Figure 10. Furthermore, the moving average of 10 samples was implemented to the measured results to reduce the signal noise further and improve the signal resolution.

By measuring the smallest observable change in the flow velocity (Figure 10), we obtained a system resolution of around 0.006 m/s at a flow velocity around 2 m/s (corresponding to a time constant of about 3.5 ms). By increasing the sensor’s flow velocity further to about 10 m/s (decreasing the time constant to about 2.5 ms), the system’s resolution decrease to about 0.1 m/s because of the sensor’s lower sensitivity. Filtering algorithms could improve the resolution of the system further, but this would decrease the response time. The system’s response time was around 150 ms (Figure 10) without filtering, and about 1.5 s using a moving average of 10 samples. Active, feedback-assisted control of the sensor’s average operating temperature also requires time after a rapid change in flow velocity and further prolongs the response time.

While the absolute accuracy was not determined, since this requires certified calibration equipment, we evaluated the repeatability of measurement results against the reference flow meter which was used to set the test flow velocities. The maximum standard deviation of the measured time constants for ten consecutive flow measurements across the entire measurement range was less than 16 μs (in total over 10 measurements were performed), which corresponds to the measured flow-velocity uncertainty of 0.014 m/s and 0.28 m/s at flow velocities of 1 m/s and 10 m/s, respectively.

### 5.2. Evaluation of the Sensor in a Constant Power Regime of Operation for Dynamic Flow Sensing

**Figure 11:**
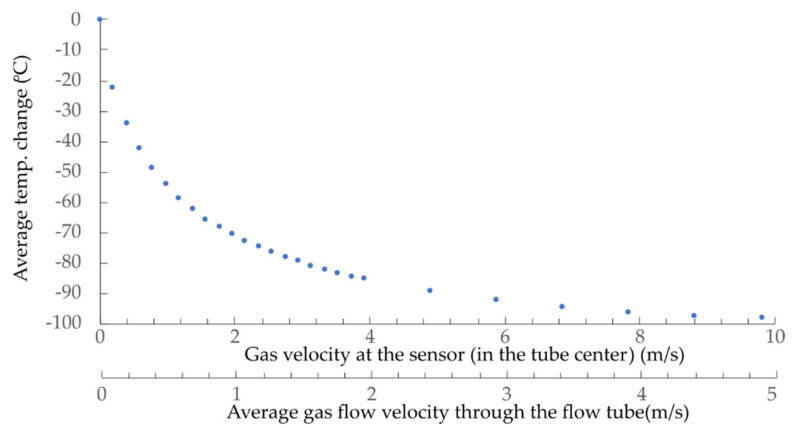
Measured average temperature change versus flow velocity at a constant heating power (9 mW).

The setup in Figure 8 was also used to test and measure the system’s static characteristics when the sensor operated in a constant power regime of operation. In this case, the sensor was heated, and the difference in the sensor’s average and ambient temperature was measured by a high-speed spectral interrogation system, as described in detail in the Signal Interrogation section (Section 4). The measured temperature difference versus flow velocity is shown in Figure 11.

The average steady state temperature of the sensing part of the sensor corresponded to about 140 °C at zero flow velocity, at an optical excitation power of 9 mW and in normal ambient conditions. All silica sensor designs might allow for operation of the sensor at even higher operating temperatures, which might allow further improvement in resolution and sensitivity.

**Figure 12:**
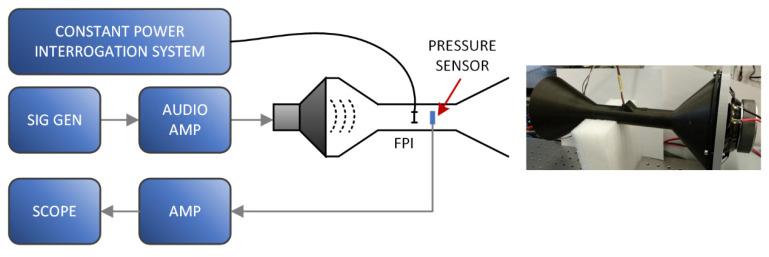
Experimental setup for the dynamic measurements.

Another experimental setup, presented in Figure 12, was prepared to investigate the sensor’s dynamic performance. A constant power regime of operation was used in this case due to the rapid system response.

A flow sensor was inserted into a conical-funnel-shaped flow tube where a speaker membrane generated a dynamic flow across the sensor. The flow tube was fixed to the speaker at the wide end, while the sensor was inserted into the tube’s narrow duct (flow velocity is higher due to the lower cross-section). The conical-funnel shape was also formed at the end of the tube to minimize back reflections of the acoustic pressure waves at the end of the tube. All gaps between the speaker and the tube were sealed to avoid any air leakage that could reduce the flow within the tube. A signal generator and an audio amplifier controlled the movement of the membrane. To ensure the control over amplitude of the flow at different frequencies, we inserted a sensitive and properly amplified reference pressure MEMS sensor near the tested optical sensor to monitor the acoustic waves’ pressure directly (acoustic (particle) velocity and pressure amplitudes of acoustic waves are proportional, i.e., *v* = *p/z*). The reference pressure sensor was required due to the low operational frequencies (2 to 20 Hz) and the use of off-the-shelf audio components. These components usually exhibit complex and strong frequency dependencies within this low-frequency band.

We exposed the sensor to dynamically changing flow velocity using the experimental setup presented in Figure 12. The average FPI temperature/spectrum variations were recorded and converted into the flow velocity using the static characteristics shown in Figure 11. Initially, the speaker excitation signal’s amplitude was constant, and flow velocities were observed at different membrane excitation frequencies of 5, 10, 20, and 80 Hz.

**Figure 13:**
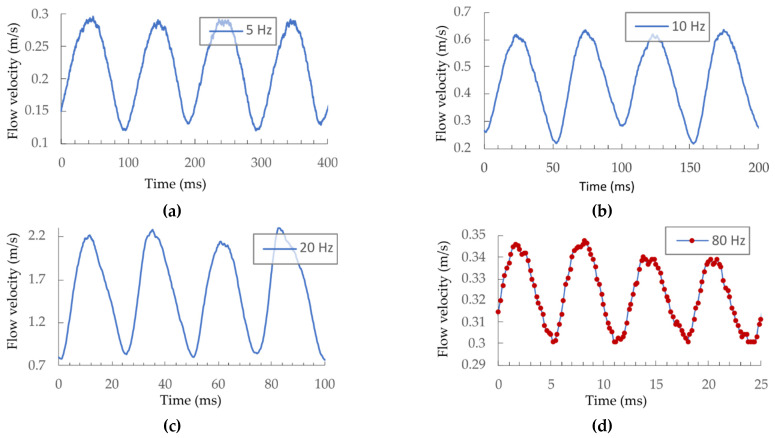
Flow velocities under dynamic changes at (**a**) 5 Hz, (**b**) 10 Hz, (**c**) 20 Hz, and (**d**) 80 Hz.

**Figure 14:**
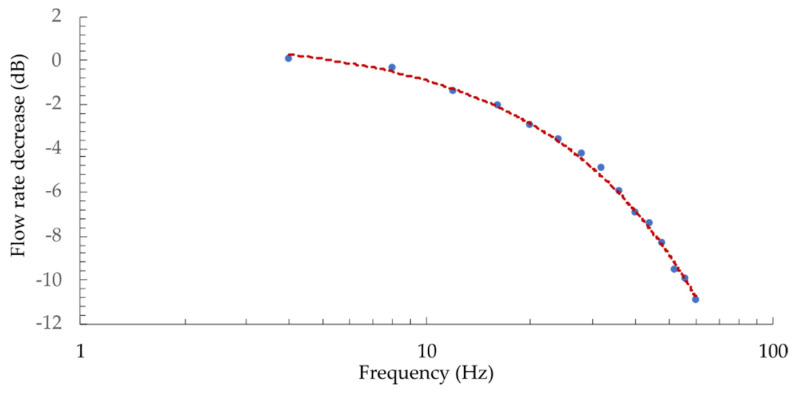
Frequency characteristics of the flow-velocity sensor obtained at a constant flow-velocity amplitude.

The acquired flow-velocity responses are presented in Figure 13. The frequency obtained by the sensor was doubled compared to the membrane excitation frequency. This was expected, since the sensor measures absolute fluid flow velocity and is not sensitive to the flow direction, which results in a response with doubled frequency (within each complete cycle, the flow direction changes and reaches two oppositely signed local peak values). Dynamic flow velocities (with higher amplitudes) were detectable at frequencies as high as 160 Hz (Figure 13d). To characterize the sensor’s dynamic properties further, we measured its frequency characteristics. During this test, the amplitude of the pressure within the flow tube was maintained constant, using the reference MEMS pressure sensor and the speaker drive current. The flow-velocity amplitude was observed at speaker excitation frequencies between 2 and 30 Hz (sensor response frequencies between 4 and 60 Hz) and converted into the dB scale (the first measurement was used as input value), as presented in Figure 14.

The numerical model predicted the corner frequency (−3 dB) at 24 Hz (the calculated time constant of 6.5 ms), while the measured results showed a corner frequency at approximately 22 Hz. The time constant changes with the flow-velocity (Figure 9), thus sensor’s bandwidth also depends on flow velocity. The sensors’ bandwidth at flow velocities around 4 m/s (time constant of 2.5 ms) would be around 64 Hz. The flow-velocity resolution of the system depends mainly on the interrogation system’s spectral resolution, which, in our case, is about ±1 pm. The resolution at lower flow velocities (higher sensitivity) is, thus, around 0.003 m/s (at 0.4 m/s), while, at higher flow velocities, it is about 0.16 m/s (at 9 m/s).

**Figure 15:**
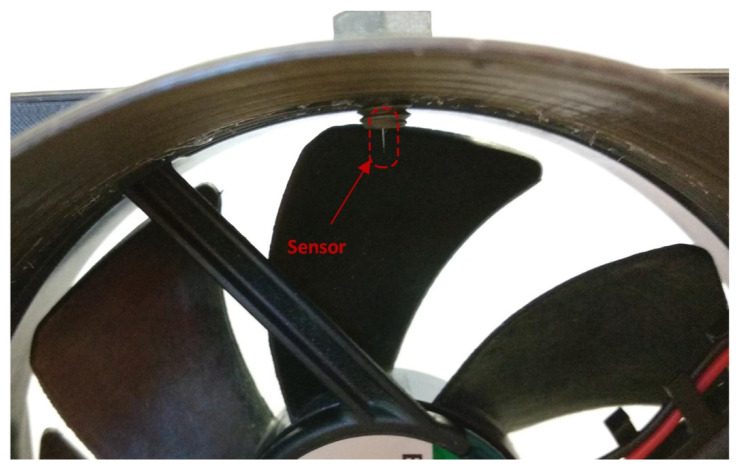
A sensor positioned in front of the fan blades to observe individual blade movement near the flow-velocity sensor.

To further demonstrate the sensor’s applicability for turbulent flow measurement, we inserted the sensor in front of a fan with seven blades, as illustrated in Figure 15.

**Figure 16:**
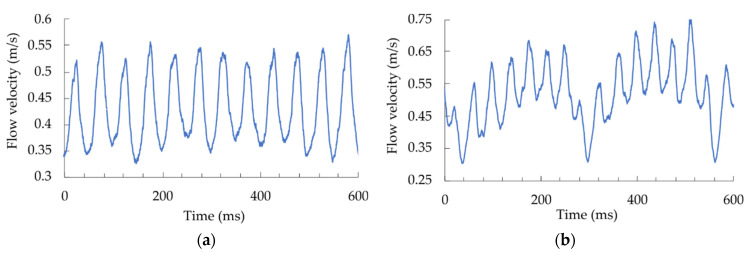
Dynamic flow changes behind rotating fan blades (7-blade fan at 180 rpm): (**a**) undamaged fan; (**b**) one blade is deformed (a small square-shaped object was attached near the tip of the blade to disturb flow through the blade).

The fan rotated at 180 rpm, while flow through at the sensor was monitored (Figure 16a).

The sensor and interrogation system acquired every pass of the rotating blades in the sensor’s vicinity. In the second example, we attached a small square-shaped object near the tip of one blade that distorted the airflow around the blade and observed the response (Figure 16b). The deformed blade is visible due to a decreased flow velocity when passing near the sensor.

## 6. Conclusions

This paper presented the design, modeling, manufacturing, and testing of a fast response time, all-fiber flow-velocity sensor. The main sensing element of the proposed sensor is an optically heated microwire with a diameter of about 16 μm; however, the core size and numerical aperture of the microwire remained the same as in a conventional single-mode fiber. This provided a microwire-sensing structure, which was optically isolated from the surrounding by silica cladding and thus resilient to potential optical contamination. The small diameter of the heated sensing element (φ16 μm) provided the sensor’s thermal time constant of about 6.5 ms in still air, a value well below any previously reported fiber-optic flow sensors (140 to 700 ms [6,7]), which was reduced further by the onset of the gas flow at the sensor. Short and flow-dependent thermal time constants provided the possibility of realizing a flow-velocity measurement system that utilized measurement of the sensor’s time constant rather than measurement of the temperature difference between the sensor and the surrounding gas. This led to a measurement configuration independent of the absolute heating power delivered to the sensor, which is essential in fiber-optic systems where precise power control, especially at remote locations, proved to be difficult and always limited. This measurement approach also resulted in a simple and cost-efficient interrogation system, which utilized only a heating laser diode, Telcom DFB diode, the detector, and the digital signal processing unit. The presented time-constant-based approach provided stable and high-resolution gas flow measurement (a resolution up to 0.003 m/s was demonstrated experimentally).

The sensor’s short response time also allowed for a dynamic flow-sensing application (including turbulence detection). The same sensor was also used to demonstrate dynamic flow sensing but in a regime where constant optical heating power was delivered to the sensor while applying high-speed spectral integration to resolve the temperature difference between the sensor and the surrounding gas. This approach provided a possibility for the dynamic measurement of flow velocities. The sensor’s bandwidth was measured experimentally and proved to be in the range of around 22 Hz at low flow velocities. With increasing the flow velocity, the bandwidth increases and allows for detection of even more rapid gas-flow-velocity fluctuations. This second system’s flow resolution depended mainly on the interrogation’s spectral resolution and was in the range of about 0.006 m/s in our specific case.

The derived semi-analytical model of the sensor is in excellent agreement with the experimental data.

The reduction of the sensor diameter could increase the dynamic performance of the sensor further. This would decrease the time constant and, thus, response times, which would allow for observation of the dynamic flow velocities at even higher bandwidths; however, this might also imply the use of shorter heating and interrogation wavelengths and single-mode fibers with smaller cores and higher numerical apertures. Both requirements might be limiting from the perspective of the components’ availability and system cost; thus, the proposed system design is likely close to an optimum that can be realized efficiently while relying on widely available components.

## Figures and Tables

**Table 1:** Parameters for the numerical model.

Parameter	Symbol	Value
Sensor (silica glass) density	*ρ*	2200 kg/m^3^
Fiber specific heat capacity	*c_s_*	730 J/kg K
Sensor radius	*r*	8 × 10^−6^ m
Fluid (air) thermal conductivity	*k_f_*	0.026 W m^−1^ K^−1^
Fluid (air) specific heat	*c_f_*	1006 J/kg K
Dynamic fluid (air) viscosity	*u_f_*	18.37 × 10^−6^ Ns/m^2^

## Data Availability

Not applicable.
